# Impact of Covid-19 on research output by gender across countries

**DOI:** 10.1007/s11192-021-04245-x

**Published:** 2022-01-27

**Authors:** Giovanni Abramo, Ciriaco Andrea D’Angelo, Ida Mele

**Affiliations:** 1 Laboratory for Studies in Research Evaluation, Institute for System Analysis and Computer Science (IASI-CNR), National Research Council of Italy, Rome, Italy; 2 Department of Engineering and Management, University of Rome “Tor Vergata”, Rome, Italy

**Keywords:** Lockdown effect, bioRxiv, Preprint depositions, Research production, Time series analysis

## Abstract

The massive shock of the COVID-19 pandemic has already shown its negative effects on economies around the world, unprecedented in recent history. COVID-19 infections and containment measures caused a general slowdown in research and new knowledge production. Because of the link between R&D output and economic growth, it is to be expected then that a slowdown in research activities will slow in turn the global recovery from the pandemic. Many recent studies also claim an uneven impact on scientific production across gender. In this paper, we investigate the phenomenon across countries, analysing preprint depositions in main repositories. Differently from other works, that compare the number of preprint depositions before and after the pandemic outbreak, we analyse the depositions trends across geographical areas, and contrast after-pandemic outbreak depositions with expected ones. Differently from common belief and initial evidence, the decrease in research output is not more severe for women than for men.

## Introduction

Following the COVID-19 outbreak in China and the Far East first, Italy and Europe shortly after, and finally the Americas, governments adopted a body of emergency measures to contrast the pandemic diffusion. Among others, mobility restrictions and social distancing caused simultaneous disruptions to both supply and demand in a globalized world economy. On the supply side, reduction of labour supply because of infections, business closures and slowdown of operations, caused a decrease in production. On the demand side, notwithstanding social safety nets introduced by governments, layoffs, loss of income, and worsened economic prospects caused a reduction in household consumption and private investment.

A rapidly growing number of studies investigate the macroeconomic effects of COVID-19 pandemic across countries, sectors in individual countries, as well as on a global scale (Pagano et al., 2020; Ludvigson et al., 2020; Baqaee & Farhi, 2020; McKibbin & Fernando, 2020). According to the World Bank, the massive shock of the COVID-19 pandemic and lockdown measures to contain it have plunged the global economy into the worst economic depression since World War II.^1^ The negative effect on economies around the world is expected to lead to a decline in per capita income in about 90% of countries in 2020 (Djankov & Panizza, 2020), not to mention the long-term social effects.

Due to containment measures, research activities, both public and private, have undergone a general slowdown as well, especially in those disciplines where the presence at work and close interaction with colleagues are necessary. “Levels of self-perceived productivity dropped, where dry lab scientists were much more likely to continue carrying out their work from home as expected (29% of dry lab scientists, but only 10% of wet lab scientists, reported “at least 80% productivity”)” (Korbel & Stegle, 2020). At many major research universities, non-essential research was halted, “in what amounts to an unprecedented stoppage of academic science in modern memory” (Redden, 2020).

It is widely accepted in economic theory that R&D spending can lead to rates of return well above those expected on standard capital investment (Deleidi et al., 2019). It is to be expected then that a slowdown in research output will in turn slowdown global recovery from COVID-19.

In this work, we undertake empirical analysis of the impact of COVID-19 pandemic on worldwide research production, across macro-geographical areas and gender. We expect that the extent of slowdown in research activities varies across countries and over time, depending on the spread of infections, the extent of social restrictions, and timing of both. Furthermore, the adoption of remote working, especially at universities and public research institutions, alongside the shutdown of schools, caused a considerable increase in the scientists’ workload at home that could impact research production differently across gender. In fact, the more extensive involvement of women in family responsibilities, mainly care for children (Schiebinger & Gilmartin, 2020), might have increased or relieved because of the presence of men at home. Specularly, men might face more distractions and an intensification of domestic responsibilities when confined to the home.

The implications of findings are twofold. First, any forecasts of the impact of research on economic recovery and growth, might be misleading if based on R&D spending during the pandemic. In fact, COVID-19 pandemic did not affect so much overall R&D spending, at least by governments (Radecki & Schonfeld, 2020), while it did affect research productivity, as we will show empirically. Second, if the pandemic unevenly affects research productivity across gender, any research performance evaluation should account for that, in order not to disfavour either gender in their careers and access to resources, and institutions with uneven gender distributions of research staff.

The first empirical studies on the effects of COVID-19 pandemic on research activities focused on the response from researchers to address health issues to minimize its impact. While findings need to be verified at a later stage of the pandemic and in the years to come, from these very first investigations we learn that the volume of publications for this topic noticeably increased (Vasconcelos et al., 2021; Li et al., 2021; Zhang et al., 2020), aswell as that of dubious and retracted research (Ioannidis et al., 2021; Pai, 2020), in fact their quality seems below the quality average of other articles in the same journals (Zdravkovic et al., 2020). Differently from previous investigations though, there seems to be a high degree of convergence between articles shared in the social web and citation counts (Kousha & Thelwall, 2020).

Soon after, the focus of scholars extended to investigate the effect of COVID-19 also on scientists’ behaviour and on research activities other than COVID-19-related research. In terms of scientists’ research behaviour, it is evident that the pandemic emergency led to substantial innovation in research collaboration and scholarly communication (Cai et al., 2021; Colavizza, 2021; Lee & Haupt, 2021). The sense of urgency that has pervaded the world scientific community has generated amounts of data sharing and scientific research collaboration at levels that have never been seen before. A speed-up of open early-stage research sharing has been reported, with a surge of depositions to such preprint archives as medRxiv and bioRxiv to foster large-scale early-stage research communication (Callaway, 2020).

The question of whether the pandemic is disproportionately hurting the productivity of female scholars has been posed and empirically confirmed in different research disciplines and from different data sources: among Italian astronomy and astrophysics researchers (Deleidi et al., 2019); among neuro-immunologists (Ribarovska et al., 2021); among corresponding authors in medRxiv, but not in bioRxiv (Wehner et al., 2020); in the physical-sciences repository arXiv and, contrary to Wehner et al. (2020), in bioRxiv as well for the life sciences (Frederickson, 2020); in 11 preprint repositories, expanding disciplinary coverage, and especially on COVID-19-related research **(**Vincent-Lamarre et al., 2020).

In a recent survey of faculty or principal investigators in the USA and Europe (Myers et al., 2020), all else being equal, female scientists reported a 5% larger decline in research time than their male peers during the Covid-19 pandemic. For scientists with at least one child five years old or younger, the decline in research time was even 17%. The authors recalled that women tended to be the primary care-givers of young children. A much vaster global survey of about 20,000 scholars confirmed findings: female academics with children reported a disproportionate reduction in research time, both relative to childless men and women and to male academics with children (Deryugina et al., 2021). Also, early journal submission data suggest that COVID-19 pandemic is disproportionally plunging women’s research production.^2^

Given that submission data are not publicly available, in the present study we recur to preprint repositories as data sources. In the past few years, there has been a significant uptake in posting preprint works in such repositories, to accelerate the diffusion of new knowledge. Considering this, and differently from other studies on the subject, we measure the variation in research production during the pandemic, by comparing research production in the pandemic period with the expected one, as extrapolated from the trends, rather than with that in previous period.

The paper unfolds as follows. In the next section we present methods and data. In the third section we present the results of the empirical analysis. The final section is devoted to the discussion and conclusions.

## Data and methods

Preprint repositories appear particularly appropriate to observe the effects of the COVID-19 pandemic and of the consequent containment measures on the production of novel scientific knowledge at a short temporal distance from its outbreak. In fact, even assuming editors’ acceptance rates unchanged, the use of a traditional bibliographic platforms (such as Scopus, WoS, Google Scholar, Dimension or the like) would in fact require a longer time window, considering the average publication time of an article in a journal, and its indexing in bibliographic repertories.

Initially, we have examined the appropriateness of a number of preprint archives to meet our objectives and methodology. For most of them, the volume of preprints is too small to assure robust elaborations. The only exceptions are arXiv, bioRxiv and medRxiv. arXiv is a free online archive and distribution service for unpublished preprints primarily for research in physics, astronomy and mathematics. bioRxiv is the equivalent in the life sciences. It was launched in 2013 by Cold Spring Harbor Laboratory, a not-for-profit research and educational institution,^3^ recently obtaining support by the Chan-Zuckerberg initiative (Callaway, 2017). First depositions occurred in November 2013. medRxiv, more focused on biomedical research and clinical medicine, was launched in 2019 by the same Laboratory, and immediately integrated in bioRxiv.

We recur to the three archives to investigate the trends of overall depositions. To delve into country- and gender-level differences though, only bioRxiv is suitable. In fact, arXiv provides authors’ affiliations of a very small share of preprints (around three percent), which makes it unsuitable for country-level analyses like the one we intend to conduct. Alongside medRxiv is too recent to be used for time series analysis, and extrapolate the trends of preprints posting.

We remind that in bioRxiv, and in general in all preprint repositories, articles are not peer-reviewed before being posted online. They undergo just a basic screening process for checking non-scientific content and plagiarism. Generally, an article may be posted prior to, or concurrently with, submission to a journal so that the authors are able to make their findings immediately available to the scientific community and receive feedback on draft manuscripts.

Before the pandemic outbreak, very few bibliometric studies had used bioRxiv as a data source. Tsunoda et al. (2019) investigated the evolution of a set of papers posted on bioRxiv and then published in academic journals. Among others, the authors found that only around 40 percent of 2013—2019 depositions are then published in peer-reviewed scientific journals. Fraser et al. (2019) investigated the citation and altmetric advantage of depositing preprints to bioRxiv. Kenekayoro (2020) recently showed that although the platform is not yet mature enough for reliable analyses, the exponential growth in preprint depositions suggests that this data source will be soon a valuable resource for discovering interesting trends on emerging or dying research fronts. Most importantly, bioRxiv provides free and unrestricted access to all preprints posted on the server. This applies also to machine analysis of the content. Metadata is made available via a number of dedicated RSS feeds and APIs resources.

For the purpose of this research, we used a wget script for retrieving all publication metadata in XML format. Data extraction took place on 16 June 2021. We retrieved all XML files related to the original deposition (version 1)^4^ of all preprints on the arXiv, bioRxiv and medRxiv servers. For bioRxiv only we then implemented a parser in Python for extracting relevant information from each XML file. More in detail, we extracted: date of deposition, doi (record digital identifier in bioRxiv), author first names and last name, author position, corresponding author, institution name and country, and subject area.

Since the full set of processed XML files from bioRxiv is deposited each month with delivery completing typically in the first days of the following month, we can be confident that the dataset contains all depositions up to 31 May 2021.

The full dataset retrieved from bioRxiv is made of 128,848 preprints, showing a quadratic growth along years up to the 2020 pandemic: 98 depositions in 2013, 845 in 2014, 1706 in 2015, 4591 in 2016, 11,191 in 2017, 20,512 in 2018, 29,171 in 2019, 41,570 in 2020 and 19,164 at 16 June 2011. The overall distribution by subject area is shown in Table 1.

**Table 1:** Share of total bioRxiv depositions by subject area

Subject area	Share (%)	Subject area	Share (%)
Neuroscience	17.1	Molecular Biology	3.4
Microbiology	8.9	Biochemistry	3.4
Bioinformatics	8.6	Immunology	3.4
Genomics	5.7	Plant Biology	3.2
Evolutionary Biology	5.5	Developmental Biology	2.9
Cell Biology	5.1	Bioengineering	2.4
Epidemiology	4.6	Systems Biology	2.3
Genetics	4.4	Animal Behavior and Cognition	1.5
Biophysics	4.3	Physiology	1.5
Ecology	4.3	Pharmacology and Toxicology	1.0
Cancer Biology	3.5	Others	2.9

For the identification of the gender of each author we queried the *Gender API* platform^5^ by the “first name” + “affiliation_country” pair. Since the level of standardization of retrieved data was not very high, some initial effort was needed for manually cleaning and reconciling fields, mainly for removing umlauts and other non-ASCII characters in first names as well as for reconciling country names.

## Results

### Spatiotemporal analysis of the pandemic impact

**Fig. 1:**
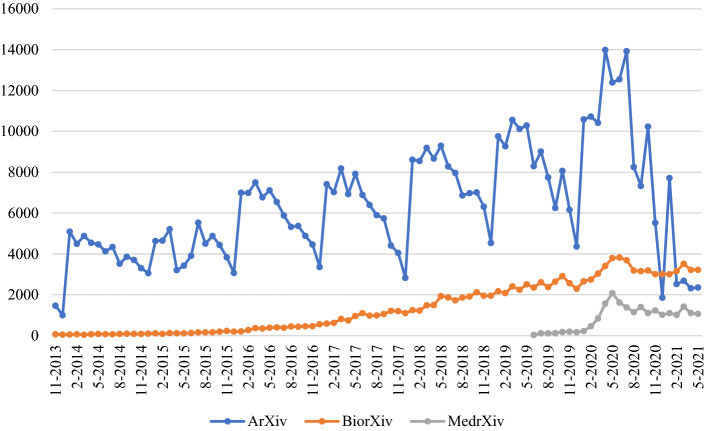
Time series of overall preprint depositions in three repositories

The number of monthly preprint depositions in the three repositories considered show quite different time patterns (Fig. 1).

**Fig. 2:**
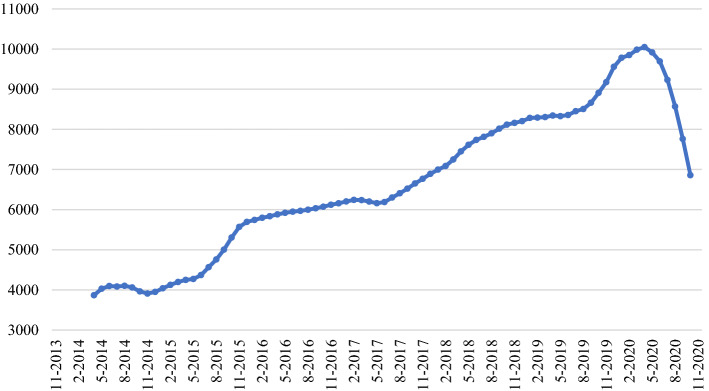
Time series of overall aRxiv preprint depositions, 12-months moving average

The plot of arXiv data shows very pronounced temporal fluctuations as compared to those for bioRxiv and medRxiv. However, all three trends present a clear discontinuity in the middle months of the 2020s. In order to better appreciate this discontinuity, the data have been seasonally adjusted in the following figures. Figure 2 shows the arXiv data, in terms of a moving average centred on 12 consecutive months. The (apparently linear) growth trend of depositions increases until April 2020: afterwards the curve shows an abrupt inversion.

**Fig. 3:**
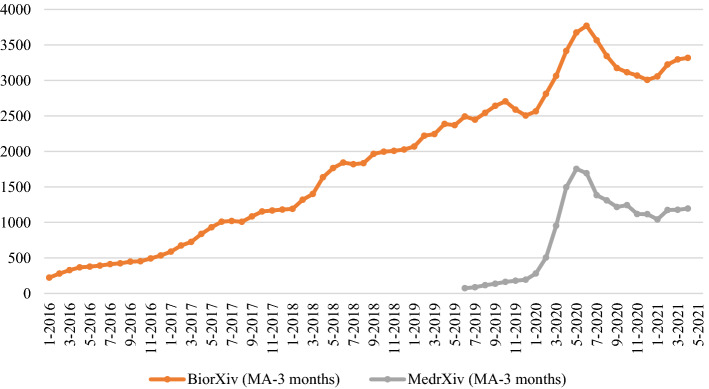
Time series of overall bioRxiv preprint depositions, 3-months moving average

The bioRxiv monthly depositions in Fig. 3 fit a non-linear growing trend up to the end of spring 2020,^6^ with a peak in June 2020. After that, we observe an abrupt inversion of the curve, indicating a disruptive effect of the pandemic on research production. A very similar trend is registered for medRxiv: launched just in 2019, the number of preprints posted in this repository grows dramatically till the peak in May 2020. Note that for bioRxiv and medRxiv, the seasonal adjustment of the data was carried out by means of a moving average over 3 months only.

To better appreciate the spatiotemporal dynamics of depositions before and after the pandemic outbreak, we stratify the data by geographical area. For this type of analysis, as mentioned above, we are forced to consider the bioRxiv depositions only. To start with, we identify the corresponding author of each preprint, the relevant affiliation and the corresponding geographical macro-area. Out of 128,848 bioRxiv preprints, 120,713 (93.7%) show at least one corresponding author. Some preprints show multiple corresponding authors, for a total of 153,231 records. 145,786 of them (95.1% out of total) are provided with affiliations which can be localized in a country and then in a geographical macro-area.

**Fig. 4:**
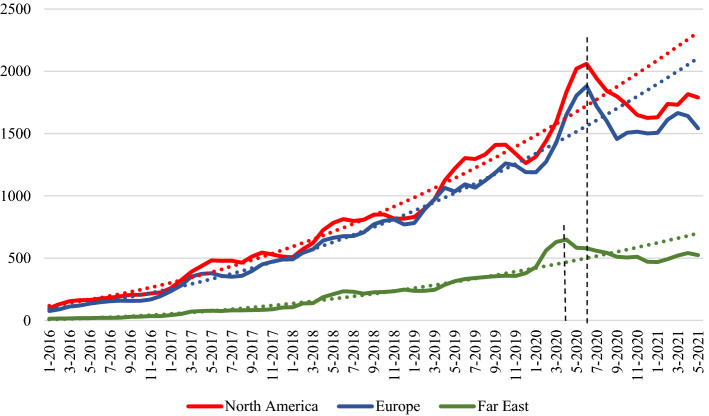
Time series of bioRxiv preprint depositions by macro-area of the corresponding author

Figure 4 shows the plots of the time series depositions in three macro-areas: Europe, North America (including Canada, USA, and Greenland) and Far East (including China, Japan, Korea and Taiwan). The dot lines represent the quadratic interpolation of the yearly moving average, as measured from November 2013 to April 2020 for the Far East, and to June 2020 for Europe and North America. In order to better visualize the curve inversion in the last period, the plot starts from 2016.

The abrupt inversion of the trend seems slightly more noticeable in Europe and North America than in the Far East. The time series reflect the timing of pandemic outbreak in the different geographical areas, occurring first in the Far East but with relatively weaker effects.

It is important to notice that the average number of corresponding authors per preprint hardly changes after the pandemic outbreak (1.27 up to June 2020; 1.28 afterwards). Therefore, all trends observed with reference to the corresponding authorships remain valid for the preprints as well.

Table 2 shows the percentage variations between observed and expected number of depositions in the period from July 2020 to May 2021. The expected number of depositions is calculated as the product of the trend by the monthly seasonality coefficients derived from the time series. We observe a drop in the number of depositions vìs-a-vìs the expected values of 15.2% at world level.^7^

**Table 2:** Observed and expected bioRxiv preprint depositions by macro-area of affiliation of the corresponding authors (July 2020–May 2021 data)

	Observed	Expected	Variation (%)	95% confidence interval*
Europe	17,264	20,031	− 13.8	[− 16.3%; + 17.2%]
North America	19,269	21,896	− 12.0	[− 14.4%; + 18.1%]
Far East	5684	6313	− 10.0	[− 30.0%; + 50.9%]
World	37,532	44,278	− 15.2	[− 10.8%; + 19.1%]

*Such interval represents the max–min variation between observed and expected values, taking into account the tails’ cut (α = 0.05)

At the macro-area level, a drop of − 13.8% was recorded for Europe, − 12.0% for the US and − 10.0% for the Far East. It should be noted that these three values are all lower than the world one, due to the different counting method. At world level, the decrease refers to the actual depositions of preprints, while for the single macro areas, we refer to the corresponding authorships that can give rise to multiple counts.

**Fig. 5:**
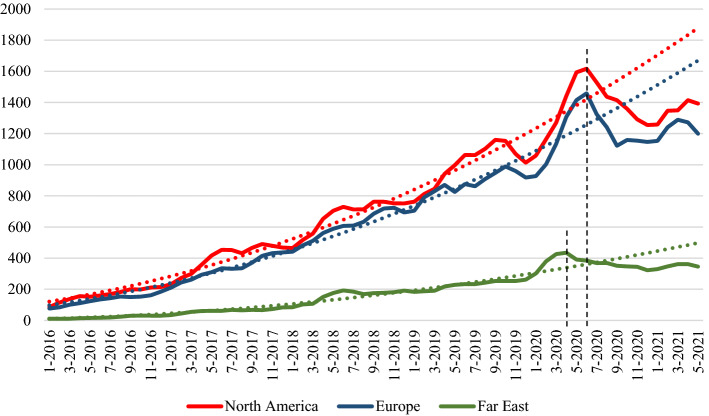
Time series of bioRxiv preprint depositions by macro-area of affiliation of the first author

If we consider the first author in place of the corresponding author, we obtain the plot of Fig. 5, showing a pattern similar to Fig. 4, the only difference being the absolute values, because on average publications report more corresponding authors than first authors.

### A gender analysis of COVID-19 impact on research production

We repeat the previous analysis further stratifying the dataset by country and gender. To discriminate gender, the first name of the author is needed alongside the country of affiliation. We discard all corresponding authors to which the *Gender API* is not able to associate a gender with an accuracy equal or above 90 percent. We are aware of the low accuracy of gender prediction for Chinese scholars (Zhao & Kamareddine, 2018); however, none of the other available resources, including NamSor Chinese API,^8^ provided higher levels of reliability than *Gender API*. The dataset for this analysis consists of 153,231 corresponding authorships. 120,946 (78.9%) are provided with a gender predicted, as said above.

**Fig. 6:**
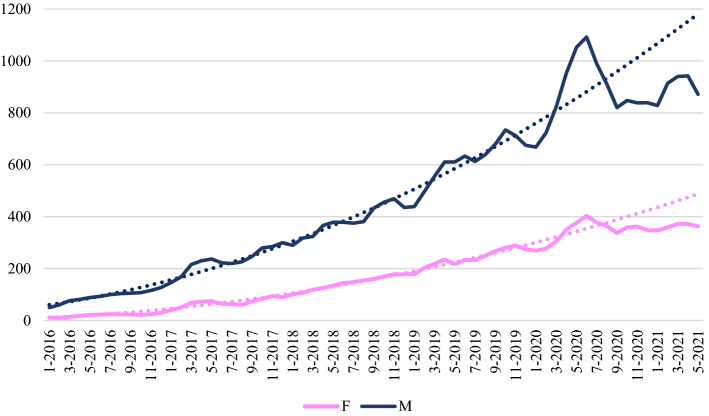
Time series of bioRxiv preprint depositions by gender of corresponding authors affiliated to European research organizations

**Fig. 7:**
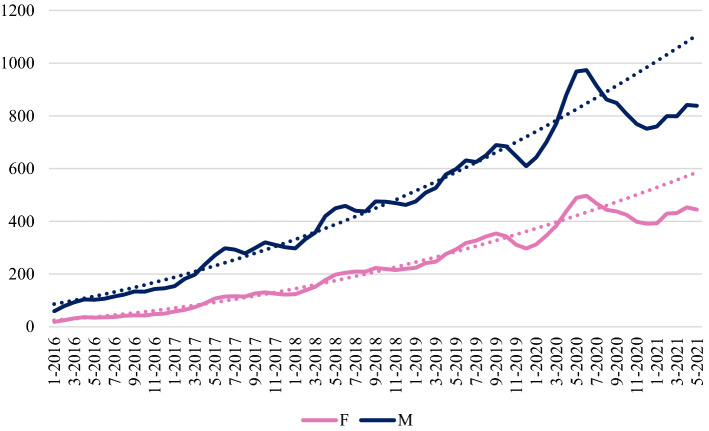
Time series of bioRxiv preprint depositions by gender of corresponding authors affiliated to North American research organizations

**Fig. 8:**
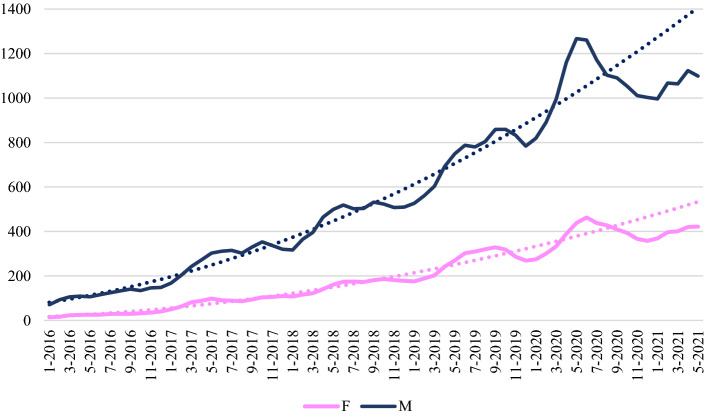
Time series of bioRxiv preprint depositions by gender of corresponding authors affiliated to Far East research organizations

Figures 6, 7 and 8 show the time series for preprints posted by corresponding authors affiliated respectively to European, North American and Far Eastern research organizations. To the eye, comparing the “expected” and “observed” curves around the pandemic outbreak, it seems that the plunge in production in all continents is much more severe for men than for women. To check whether this is also true in relative terms, we conducted a more in-depth analysis taking into account also the seasonality of depositions. Results are shown in Table 3, and reveals that for Europe the plunge in depositions is similar for women (− 13.4%) and for men (− 13.6%). The same occurs in North America (− 11.9% vs. + 12.0%). In the Far East instead, women experienced a worse decrease in depositions (− 16.2%) with respect to men (− 9.3%). In Europe, women accounted for 35.7% of total corresponding authorships up to June 2020, compared with 40.9% in the following 11 months. North America also showed a positive trend, 33.4% before vs 37.6% after. In contrast, at the turn of June 2020, in the Far East the incidence of women among the corresponding authors of bioRxiv preprints declined, from 22.1% to 21.5%.

**Table 3:** Observed and expected bioRxiv preprint depositions by macro-area and gender of corresponding authors (July 2020–May 2021 data)

	Gender	Observed	Expected	Variation (%)
Europe	F	3982	4596	− 13.4
	M	9736	11,268	− 13.6
North America	F	4418	5012	− 11.9
	M	11,743	13,350	− 12.0
Far East	F	709	846	− 16.2
	M	3293	3632	− 9.3

We delve country level analyses, considering the U.S., China, and the top eight European countries per number of depositions. Findings in Table 4 confirm the imbalance showed in Table 3 in the U.S., where female scientists reduced the output of their research activities at a rate similar to that of their male colleagues, − 14.2% versus − 14.5%. The same occurs in China: − 15.9% for females versus − 16.4% for males. In Europe, contrasting evidences emerge. In France, Netherlands and Switzerland it is women who are hurt more, while in the other countries the opposite holds true. In Germany, the decrease registered for men (− 11.7%) is almost three times that for women (− 4.1%). Quite surprisingly, in Spain female scientists raised their depositions with respect to the expected ones, by 14.8 percent, while males decreased theirs by 13.8 percent. In Italy, Sweden and U.K., gender differences are hardly noticeable. It must be said that the more fine-grained the analysis the less robust the results, because of the lower number of observations. Nevertheless, what emerges at continental level is often untrue at country level, where the interplay of different containment measures, women’s role in society, and family-related infrastructure unveil quite different realities. For sure, there are countries where women have shown greater resilience and adaptability than men to the new working conditions brought about by the measures taken to contrast the pandemic, and/or have benefited from the greater male presence at home to share family responsibilities.

**Table 4:** Observed and expected bioRxiv preprint depositions by gender of the corresponding authors in the U.S., China, and the top eight European countries per number of depositions (September–November 2020 data)

Country	Gender	Observed	Expected	Variation (%)	M-F variation (%)
United States	F	4082	4760	− 14.2	− 0.30
	M	10,839	12,676	− 14.5	
China	F	498	592	− 15.9	− 0.50
	M	1667	1994	− 16.4	
France	F	498	707	− 29.6	9.40
	M	1151	1442	− 20.2	
Germany	F	773	806	− 4.1	− 7.60
	M	2159	2446	− 11.7	
Italy	F	169	181	− 6.6	− 1.50
	M	424	461	− 8.1	
Netherlands	F	202	271	− 25.6	10.10
	M	534	632	− 15.5	
Spain	F	247	215	14.8	− 28.60
	M	487	565	− 13.8	
Sweden	F	171	185	− 7.8	1.10
	M	397	426	− 6.7	
Switzerland	F	255	292	− 12.8	10.30
	M	781	801	− 2.5	
United Kingdom	F	1085	1362	− 20.4	− 0.60
	M	2521	3193	− 21.0	

**Fig. 9:**
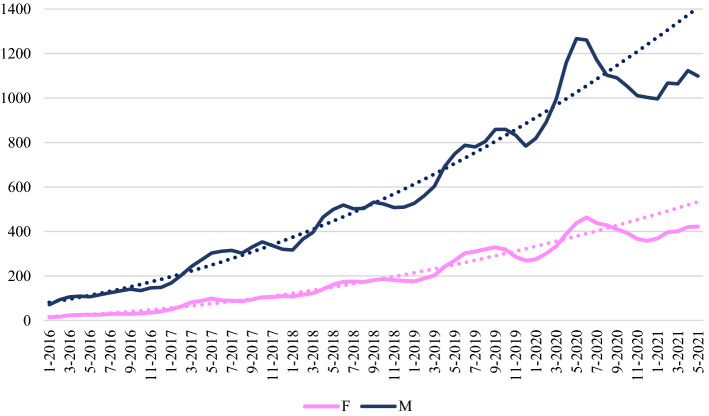
Time series of fractional output of bioRxiv preprints of North American authors, by gender

Finally, we investigate if and to what extent results of the analysis are sensitive to the choice of considering, as observation units, the corresponding authors. To do so, we consider all the authors of a publication and assign to each one a fraction equal to the reciprocal of the number of total co-authors. The 128,848 bioRxiv preprints give rise to a total of 954,274 authorships. Using Gender-API we were able to attribute a gender to 764,728 of these records, which is 80.1% of the total. Then we calculate the fractional output of males/females of a country/continent as the sum of their fraction for each publication they authored. This fraction was calculated by considering as denominator, for each publication, the number of co-authors tagged with a gender. As an example, Fig. 9 shows the plot of the data for North America. Beyond the differences in the absolute value of the data, the trend shown is absolutely similar to that reported in Fig. 7. The same holds true for other macro areas. Hence, we can conclude that the results obtained with the analysis referred to the corresponding authors are robust with respect to other choices about the unit of observation of the analysis and, in particular, with respect to the choice of considering all co-authors of each publication in the dataset.

## Conclusions

The human impacts of COVID-19 infections, and pandemic-related limitations and impediments are vast. These include disruptions to researchers that, as we showed, differ by nation and gender. In this work, we have investigated the impact of pandemic on research output. Results confirm what was expected, that is an abrupt inversion in preprint depositions, following the pandemic outbreak, consistent in timing across geographical areas: in China and the Far East first, followed by Europe and North America (and more noticeably).

Contrary to what most people might expect, and early studies have announced, that female scientists are hurt more by pandemic due to the increase in family care workload, we could observe that the plunge in production is not more severe for women than for men.

Only in the Far East, women experienced a worse decrease in depositions with respect to men. Both in Europe and in North America the share of women among corresponding authors of bioRxiv preprints shows a significant increase after June 2020, the month that marks the discontinuity due to the pandemic event. In fact, at country level, important differentiations emerge. In US and China female and male scholars reduced their research output at a similar rate. In Europe, contrasting evidences emerge. In some countries (France, Netherlands and Switzerland) women are hurt more than men; in others (Germany and Spain) the opposite holds true, while in such countries as Italy, Sweden and U.K. gender differences are hardly noticeable.

The important lesson to be learnt is that world level analyses often hide significant differences across countries, especially when country-specific variables play a significant role in determining the outcomes. The interplay of different containment measures, women’s role in society, and family-related infrastructure unveil quite different realities. This would require further investigation by scholars knowledgeable about the country under observation. As for Italy, results can be only partly explained: here women’s relative share of involvement in family responsibilities, mainly care for children but also for parents and parents-in-law, is more extensive than in average EU countries. Only 24 percent of the children go to kindergarten,^9^ not allowing parents to be full-time occupied (Istat, 2019). Percent of population ages 65 and older (often associated with high levels of morbidity) is 24 in Italy, among the highest in the world (PRB-Population Reference Bureau, 2020). According to a recent survey, the majority of Italians totally agreed on the statement: “The most important role of a woman is to take care of her home and family”. However, we believe that women have shown greater resilience and adaptability than men to the new working conditions. After the first phase, the continuation of containment measures in the second wave may even have benefited them by increasing male involvement in family responsibilities.

Findings might be of interest to scholars in scientometrics, in the economics of innovation, and in sociology. The pandemic effects on research can inform policy makers when dealing with economic forecasts, gender equality issues, research evaluation exercises, and the assessment of the effectiveness of relevant policies and initiatives. The revealed pandemic effect on academic research output adds to a worrying phenomenon concerning the returns on R&D investment: a number of scholars have argued that good ideas are becoming harder to find and the productivity of research may be stagnating (Bloom et al., 2020; Boeing & Hünermund, 2020). If correct, the long-term economic consequences of such a development would be many and significant.

The results of the analyses proved to be robust with respect to the choice of the unit of observation (corresponding authors, first authors or all authors in the byline). Nevertheless, we appreciate several limitations embedded in our study. The field of analysis is limited to the life sciences, therefore findings and conclusions cannot be generalized to other disciplines. Data extraction was conducted during the pandemic, whose expiration is hopefully expected to occur in the next few months. Therefore, the extent of the effects that we tried to grasp is to be confirmed by future updates. Specifically, we will probably never see the consequences of the slowdown in scientific production caused by COVID-19 pandemic, in terms of published articles, as journal editors can simply raise their acceptance rates to keep the volumes unchanged. Most likely, we will witness lower average quality of publications. Evidence of that can be assessed in the years to come. Editors though might give now a precious contribution to scholars in the field by providing them with data on submission variations after the pandemic outbreak.

Future research might also entail investigation on the pandemic impact on research collaboration behaviour. It is to be expected in fact that intra-muros collaborations must have lost their advantage over extra-muros, as physical presence and personal contacts were inhibited by containment measures.

## Footnotes

1 https://www.worldbank.org/en/news/press-release/2020/06/08/covid-19-to-plunge-global-economy-into-worst-recession-since-world-war-ii, last accessed 25 November 2021.

2 https://www.insidehighered.com/news/2020/04/21/early-journal-submission-data-suggest-covid-19-tanking-womens-research-productivity, last accessed 25 November 2021.

3 https://www.bioRxiv.org/about-bioRxiv.

4 Authors can deposit a revised version of an article at any time prior to its formal acceptance by a journal.

5 https://Gender.API.com/ last accessed 25 November 2021.

6 The diagram shows the quarterly moving averages, to filter chance fluctuations.

7 We mean the whole world, not limited to the sum of Europe, North America and Far East.

8 https://chinese-names.app/gender, last accessed 25 November 2021.

9 Pandemic containment measures spared kindergarten, which remained open most of the pandemic period.
